# Characterizing Interactions Between Small Peptides and Dimethyl Sulfoxide Using Infrared Spectroscopy and Computational Methods

**DOI:** 10.3390/molecules29245869

**Published:** 2024-12-12

**Authors:** Aneta Panuszko, Przemysław Pastwa, Jacek Gajewski, Piotr Bruździak

**Affiliations:** Department of Physical Chemistry, Gdańsk University of Technology, Narutowicza 11/12, 80-233 Gdańsk, Poland; s185357@student.pg.edu.pl (P.P.); s189068@student.pg.edu.pl (J.G.); piotr.bruzdziak@pg.edu.pl (P.B.)

**Keywords:** dimethyl sulfoxide, peptide hydration, water, FTIR spectroscopy, DFT calculations

## Abstract

This study provides a comprehensive analysis of the interactions between dimethyl sulfoxide (DMSO) and two small peptides, diglycine and *N*-acetyl-glycine-methylamide (NAGMA), in aqueous solutions using FTIR spectroscopy and density functional theory (DFT) calculations. ATR-FTIR spectroscopy and DFT results revealed that DMSO does not form direct bonds with the peptides, suggesting that DMSO indirectly influences both peptides by modifying the surrounding water molecules. The analysis of HDO spectra allowed for the isolation of the contribution of water molecules that were simultaneously altered by the peptide and DMSO, and it also explained the changes in the hydration shells of the peptides in the presence of DMSO. In the DMSO–diglycine system, DMSO contributes to the additional strengthening of water hydrogen bonds in the reinforced hydration sphere of diglycine. In contrast, DMSO has a more moderate effect on the water molecules surrounding NAGMA due to the similarity of their hydration shells, leading to a slight weakening of the hydrogen bonds in the NAGMA hydration sphere. DFT/ONIOM calculations confirmed these observations. These findings demonstrated that DMSO influences peptide stability differentially based on their structural characteristics.

## 1. Introduction

It is well established that the proper structure of proteins is crucial for their biological function and activity [1]. Since the native structure can be modulated by interactions with water molecules and other co-solutes [2,3], investigating these interactions is vital for comprehending the fundamental principles that underlie protein stability, folding, and functionality. Such insights hold particular importance in disciplines like biochemistry, pharmacology, and biotechnology [4]. Therapeutic proteins, compared to small-molecule drugs, are typically more costly to produce and exhibit shorter shelf lives. This is largely because they must remain in stable aqueous solutions over the duration of their use [5,6]. The pharmaceutical industry, therefore, places a high priority on strategies to stabilize these proteins in solution, prolonging their functional state. However, despite decades of research, the exact role of co-solutes and the molecular mechanisms by which they influence protein stability remain somewhat elusive [7,8]. Theories on protein–co-solute interactions typically fall into two main categories: direct [9,10] and indirect interactions [11,12,13,14]. Direct interactions involve the co-solutes binding to specific sites on the protein surface, potentially changing the protein’s conformation and hydration. On the other hand, indirect interactions, known as preferential hydration, influence the water molecules surrounding the protein rather than binding directly to it. These mechanisms are not mutually exclusive and can occur simultaneously, contributing to the overall effect of co-solutes on proteins [6,15].

Due to the inherent complexity of proteins, researchers frequently employ simplified molecular models, such as *N*-methylacetamide (NMA), 2-acetamido-*N*-methylacetamide (NAGMA), small peptides like diglycine, and other dipeptides or tripeptides, which offer experimental and computational accessibility due to their manageable size and peptide bond similarity to proteins [16,17].

**Figure 1:**
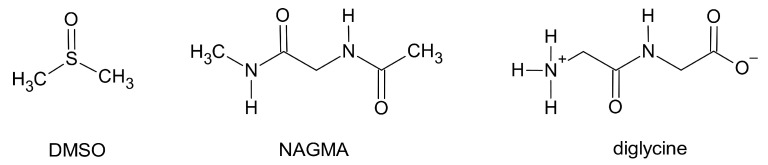
Structural formulas of DMSO, NAGMA and diglycine in aqueous solutions.

Dimethyl sulfoxide (DMSO, see the structure in Figure 1) exhibits unique properties that make it a widely used co-solvent in biochemical and pharmaceutical research. Thanks to its ability to dissolve both polar and nonpolar molecules, it can be used to enhance the solubility of compounds with low solubility in water [4,18]. Additionally, DMSO is renowned for its ability to penetrate biological membranes, making it an effective carrier for drug delivery [19,20]. In protein studies, DMSO is frequently employed to stabilize, destabilize, solubilize, crystallize, and denature proteins [21]. The influence of DMSO on protein stability is not fully understood; although, some studies indicate that at low concentrations, DMSO stabilizes the folded state of proteins through preferential hydration [22]. However, as the DMSO concentration increases, its interaction shifts from preferential hydration to preferential binding with proteins, resulting in structural changes and unfolding [20,21]. This phenomenon can be attributed to unfavorable interactions between DMSO and the polar surface of the native protein. And at higher concentrations, DMSO increases binding to hydrophobic and aromatic side chains, inducing protein unfolding. To further understand how DMSO interacts with protein backbones and side groups, various experimental and computational studies have been conducted using short peptides as model substances. For instance, studies on the peptide *N*-acetyl-leucine-methylamide (NALMA) [23] suggest that DMSO causes peptide dehydration due to interactions with its aliphatic side chain. Similarly, studies on NMA reveal that DMSO replaces water molecules at the amide bond, weakening hydrogen bonds between NMA and water while forming strong hydrogen bonds between the oxygen of DMSO and the amide hydrogen of NMA. Despite these studies, the exact mechanism by which DMSO influences protein solubility and conformational stability is still not fully defined.

In our previous work [11], using FTIR spectroscopy and computer simulations, we thoroughly characterized the hydration shells of diglycine and NAGMA and demonstrated the differences between them. Diglycine, composed of two glycine residues, is highly hydrophilic, allowing strong hydrogen bonding interactions with water due to its ability to form zwitterions. In contrast, NAGMA with methyl groups at both ends primarily exhibits hydrophobic hydration around its methyl groups and creates fewer direct interactions with water. For this reason, NAGMA is suitable as a model for hydrophobic regions of proteins, while diglycine serves as a model for proteins with polar side chains. The structural formulas of both peptides are shown in Figure 1.

In this work, we investigate the influence of DMSO on two simple model peptides: diglycine and NAGMA, by means of Fourier transform infrared spectroscopy (FTIR). Attenuated total reflectance FTIR provided insights into the interactions of DMSO with peptides in aqueous solutions. Transmission mode spectroscopy was used to characterize the interactions between the hydration shells of the peptides and DMSO. The applied method of spectral data analysis allowed for the isolation of the contribution of water simultaneously influenced by both the peptide and DMSO, as well as the determination of the structural and energetic properties of this water population. The spectral findings were supported by calculations based on density functional theory (DFT).

## 2. Results and Discussion

### 2.1. Direct Interactions Between Model Peptides and DMSO–ATR-FTIR Studies

**Figure 2:**
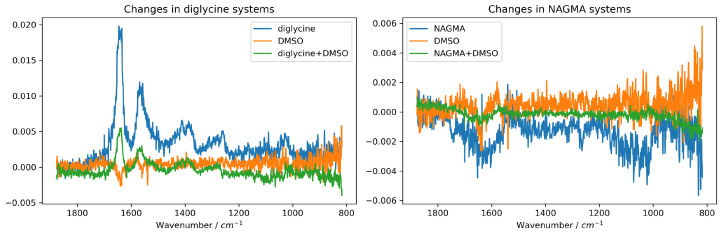
Differential ATR-FTIR spectra in the diglycine–water–DMSO systems (**left panel**) and NAGMA–water–DMSO systems (**right panel**). The blue spectra indicate differences caused by the increase in dipeptide concentration in the binary system with water alone; the orange spectra indicate such changes in the DMSO–water system; the green spectra indicate analogous changes in the ternary systems. The noise is due to the very low level of the differential signal.

The difference spectra in the ternary systems diglycine–water–DMSO and NAGMA–water–DMSO exhibit certain features that indicate changes in the oscillatory structure of the system components caused by changes in the relative concentrations of the solutes (see Figure 2 and Figure S1 for an illustration of producing such difference spectra). Although the differential bands in these spectra, resulting from shifts in absorption changes in their intensity, may at first glance suggest the formation of interactions between DMSO and diglycine or NAGMA, they should be compared with similar difference spectra determined for the pure solutes in water systems. In both ternary systems, the changes in the difference spectra are very similar to the difference spectra corresponding to pure diglycine or NAGMA. There are no significant differences between the corresponding difference spectra, except the overall intensity. In the case of direct diglycine–DMSO or NAGMA–DMSO interactions, changes in the vibration frequencies of the S=O bonds of DMSO or the N–H and possibly C=O bonds of the peptides would be expected, yet the absence of such shifts thus excludes the formation of strong and direct diglycine–DMSO or NAGMA–DMSO interactions. It is also important to note the very low intensity of the observed difference signals, especially in the case of the NAGMA–water–DMSO system, which reinforces the statement about the lack of direct interactions between the solutes. Therefore, any interactions in both systems should be mediated by water molecules. Hence, in the next step, the HDO difference spectra method will be used to search for differences in the structure of water in the ternary systems.

### 2.2. FTIR Investigation the Water Structure Around Peptides in the Presence of DMSO

The method of affected HDO spectra allows for a relatively simple characterization of solute hydration shells in terms of their energy and structure. The spectra of semi-heavy water are easier to interpret than those of pure H_2_O or D_2_O, due to the absence of certain coupled vibrations resulting from the identical masses of two vibrating and neighboring OH or OD bonds. This method was further used to characterize and compare the properties of water around DMSO, diglycine, NAGMA, and their ternary systems.

#### 2.2.1. Experimental vs. Theoretical Spectra of Affected Water in Peptide–DMSO Systems

The spectral contours of water affected by a peptide (NAGMA or diglycine) and DMSO in ternary solutions (so-called experimental affected water spectra, ε_exp._) for various molar fractions of DMSO to peptide are illustrated in Figure S2a,b. These spectra contain information about the interactions between the hydration spheres of both solutes in the aqueous solution. An excellent reference for analyzing these spectra are the so-called theoretical spectra of affected water, ε_theor._, (Figure S3a,b) which represents a hypothetical situation in the solution where each solute individually affects the water, but they or their hydration spheres do not interact with each other. We constructed them from the spectral contours of water affected by the pure components: peptide, ε_peptide_, (Figure S2a,b) and DMSO, ε_DMSO_, (Figure S4a) taking into account the theoretical numbers of water molecules affected by them (N_peptide,theor._ and N_DMSO,theor._, see Table S3 in Supplementary Materials):

(1) $$\varepsilon_{\mathrm{theor}.}=\frac{N_{\mathrm{DMSO},\mathrm{theor}.}\;\cdot \;\varepsilon_{\mathrm{DMSO}}+N_{\mathrm{peptide},\mathrm{theor}.}\;\cdot \;\varepsilon_{\mathrm{peptide}}}{N_{\mathrm{DMSO},\mathrm{theor}.}+N_{\mathrm{peptide},\mathrm{theor}.}}$$

The experimental and theoretical affected water spectra for the diglycine–DMSO and NAGMA–DMSO systems, along with the spectra of water affected by the pure components (diglycine, NAGMA, DMSO, and water), were converted using Equation (3) into the interatomic oxygen–oxygen distance distribution functions, $P(R_{OO})$. The results of the performed transformations are shown in the Supplementary Materials (Figures S2c,d, S3c,d and S4b). The parameters of these spectra and the oxygen–oxygen intermolecular distance distribution functions are summarized in Tables S1 and S2 in the Supplementary Materials.

We can observe very subtle differences between the parameters (such as the band position at the maximum and the band position at gravity center, as well as the oxygen- -oxygen intermolecular distances correlated with them) of the experimental and theoretical water affected spectra in both the diglycine-–DMSO and NAGMA–DMSO systems (Table S1 and Table S2, respectively) in the entire range of DMSO concentrations studied. This indicates that the mutual contact of the hydration shells of both solutes is only slight.

More information on the contact of the hydration shells of both solutes in these systems can be obtained by comparing the values of the number of affected water molecules in the experimental spectrum (*N*_exp._) and theoretical spectrum (*N*_theor._), namely by calculating the difference between them: Δ*N* = *N*_exp._ – *N*_theor._ The corresponding numbers of affected water molecules and the differences between them (Δ*N*) are presented in Table S3 in the Supplementary Materials. A negative value of Δ*N* points out that the hydration spheres of both solutes overlap, resulting in some affected water molecules belonging simultaneously to the hydration spheres of both solutes, i.e., they are shared water molecules in the hydration shells of both solutes. On the other hand, a positive value of Δ*N* indicates that additional water molecules are affected as a result of the contact between the hydration spheres of both solutes. In this case, we can consider the following scenarios: (1) additional water molecules originate from the perturbation of water molecules in the hydration spheres of the solutes, which were not considered affected before the contact of the hydration spheres of both solutes; (2) between the hydration spheres of both solutes there are additional water molecules connecting both hydration spheres, the so-called bridging water molecules, which come from bulk water.

Positive values of Δ*N* (approximately +2.0) recorded for the diglycine–DMSO systems across the entire range of molar ratios of DMSO to diglycine suggest that the contact of the hydration spheres of both solutes causes the perturbation of an additional two water molecules. In the case of the NAGMA–DMSO system, we can observe differences in Δ*N* values depending on the solution composition. For a molar ratio of DMSO to NAGMA of 1:1, the negative value of Δ*N* (i.e., −0.7) indicates an overlap of the hydration spheres of the peptide and DMSO. However, for molar ratios of DMSO to NAGMA from 2.0 to 5.0, positive Δ*N* values were obtained, increasing with the molality of DMSO. This means that as the molality of DMSO increases, the number of excess affected water molecules in the system also increases (up to two water molecules).

#### 2.2.2. Analysis of the Spectral Shares of Affected Water in the Experimental Spectra in Peptide–DMSO Systems

**Figure 3:**
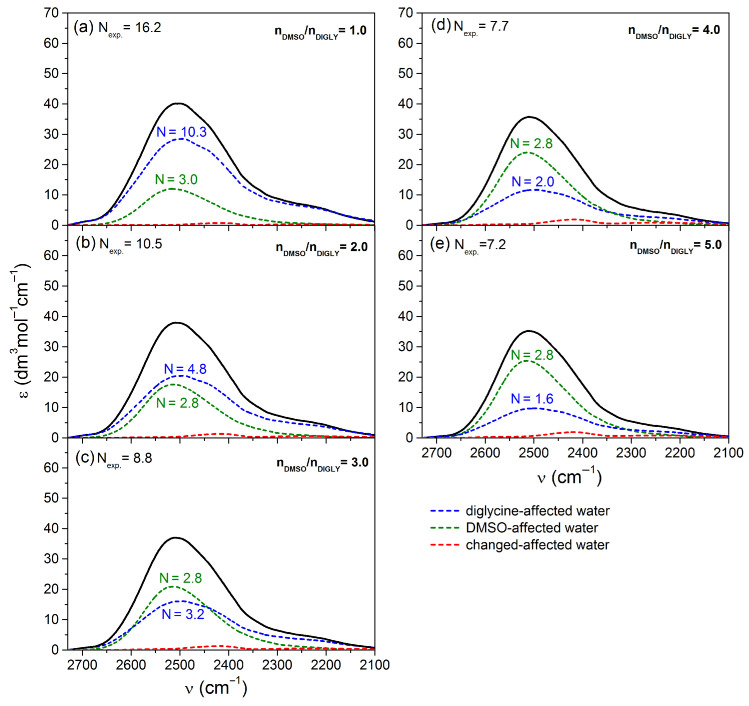
The spectra of water affected by diglycine and DMSO in ternary mixtures (experimentally affected spectra, black lines, from Figure S2a) for different molar ratios of DMSO to diglycine with separated contributions of water under the simultaneous influence of the diglycine and DMSO (i.e., changed-affected water) and contributions of water affected by pure components: diglycine-affected water and DMSO-affected water, along with the corresponding numbers of affected water molecules.

**Figure 4:**
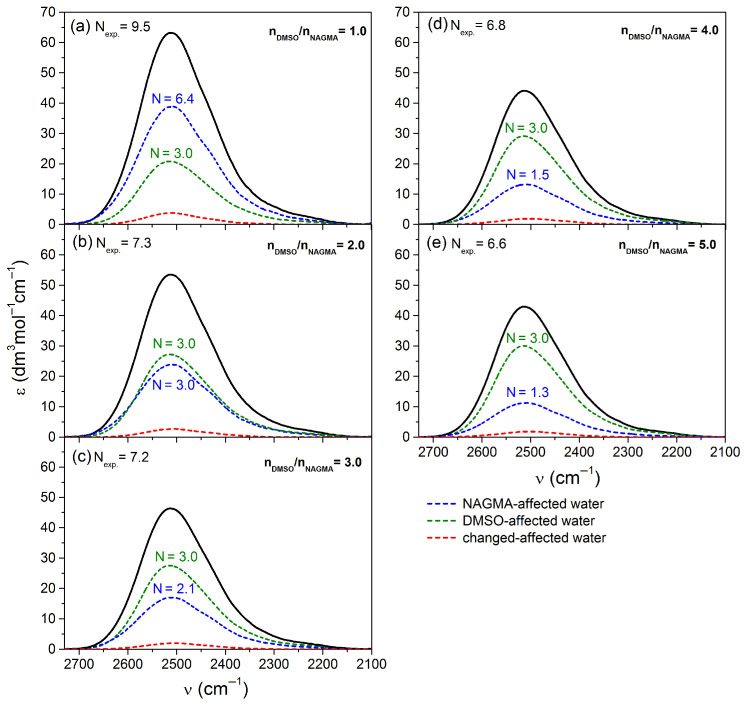
The spectra of water affected by NAGMA and DMSO in ternary mixtures (experimental affected spectra, black lines from Figure S2b) for different molar ratios of DMSO to NAGMA with separated contributions of water under the simultaneous influence of the NAGMA and DMSO (i.e., changed-affected water) and contributions of water affected by pure components: NAGMA-affected water and DMSO-affected water, along with the corresponding numbers of affected water molecules.

The spectral contributions of water molecules influenced concurrently by a peptide and DMSO (so-called changed-affected water) were extracted from the experimental affected water spectra using our fitting procedure [24]. In parallel, we determined the spectral contributions of water affected by each solute independently (i.e., water affected by pure components): peptide-affected water and DMSO-affected water, as well as the respective numbers of affected water molecules. The findings from this analysis are illustrated in Figure 3 and Figure 4, for the diglycine–DMSO and NAGMA–DMSO systems, respectively.

By analyzing the number of affected water molecules for the spectral contributions of the pure components (DMSO and peptide) in the experimental spectra (as depicted in Figure 3 and Figure 4) and theoretical spectra of affected water (Table S3), we can ascertain the number of changed-affected water molecules within the hydration sphere of each solute as a result of the interactions between the hydration spheres of both solutes. As we can see in both systems, the values of *N* corresponding to the spectral share of DMSO-affected water in the experimental spectra are either identical (in the case of the NAGMA–DMSO system) or nearly identical (for the diglycine–DMSO system) to those observed in the theoretical spectra. This indicates that the hydration sphere of DMSO remains unaffected by interactions with the peptide’s hydration sphere across the entire range of DMSO concentrations studied. The high stability of the DMSO hydration sphere is ensured by strong interactions of water molecules and the sulfonic oxygen atom, thereby maintaining a common network of water–water hydrogen bonds around the solute in this group [25]. Conversely, the discrepancies in the *N* values for the spectral contribution of water affected by peptides between the experimental and theoretical spectra imply that the water surrounding DMSO alters the properties of affected water molecules within the hydration spheres of the peptides.

#### 2.2.3. Structural and Energetic Analysis of Changed-Affected Water Molecules

The spectral shapes of the changed-affected water spectra in the diglycine–DMSO (from Figure 3) and NAGMA–DMSO systems (from Figure 4) were transformed using Equation (3) into oxygen-oxygen distance distribution functions, $P(R_{OO})$ (Figure S6 in the Supplementary Materials). The main spectral parameters of changed-affected water for different molar ratios of DMSO to diglycine in peptide–DMSO systems, as well as water affected by pure components and bulk water, along with the intermolecular oxygen–oxygen distances, are presented in Table 1. Additional spectral parameters of HDO are available in the Supplementary Materials (Tables S1 and S2).

The structural and energetic state of water molecules with changed properties in the peptide’s hydration spheres can be described by comparing the values of the gravity center of the OD bands, $\nu_{OD}^{g}$ (the mean energy of water hydrogen bonds) and the mean oxygen–oxygen distances between water molecules, $R_{OO}^{g}$ in the changed-affected water with the corresponding values of the peptide hydration water (Table 1). The shift in the values of the $\nu_{OD}^{g}$ and the $R_{OO}^{g}$ between water molecules with changed properties in the diglycine–DMSO system towards lower values compared to those for diglicine-affected water (Table 1) indicates that water molecules in the changed-affected water form significantly stronger hydrogen bonds than in the hydration sphere of diglycine (i.e., without DMSO), across the entire range of DMSO composition. This means that the interaction between the hydration spheres of diglycine and DMSO causes the strengthening of the water structure around the peptide relative to the peptide hydration shell. Moreover, the comparable values of $\nu_{OD}^{g}$ and $R_{OO}^{g}$ in the changed-affected water for all analyzed molar ratios of DMSO to peptide suggest that the DMSO concentration does not influence the peptide’s hydration shell.

In contrast, for the NAGMA–DMSO system (Table 1), the hydrogen bonds between water molecules in the changed-affected water are only slightly weaker than the water–water hydrogen bonds around the NAGMA peptide, across the entire range of DMSO concentrations studied. Only a little influence of DMSO concentration on the mean values ($\nu_{OD}^{g}$ and $R_{OO}^{g}$) in the changed-affected water can be observed. However, the effect of DMSO concentration is evident in the most probable values ($\nu_{OD}^{o}$ and $R_{OO}^{o}$), which decrease with increasing DMSO concentration in this system.

**Figure 5:**
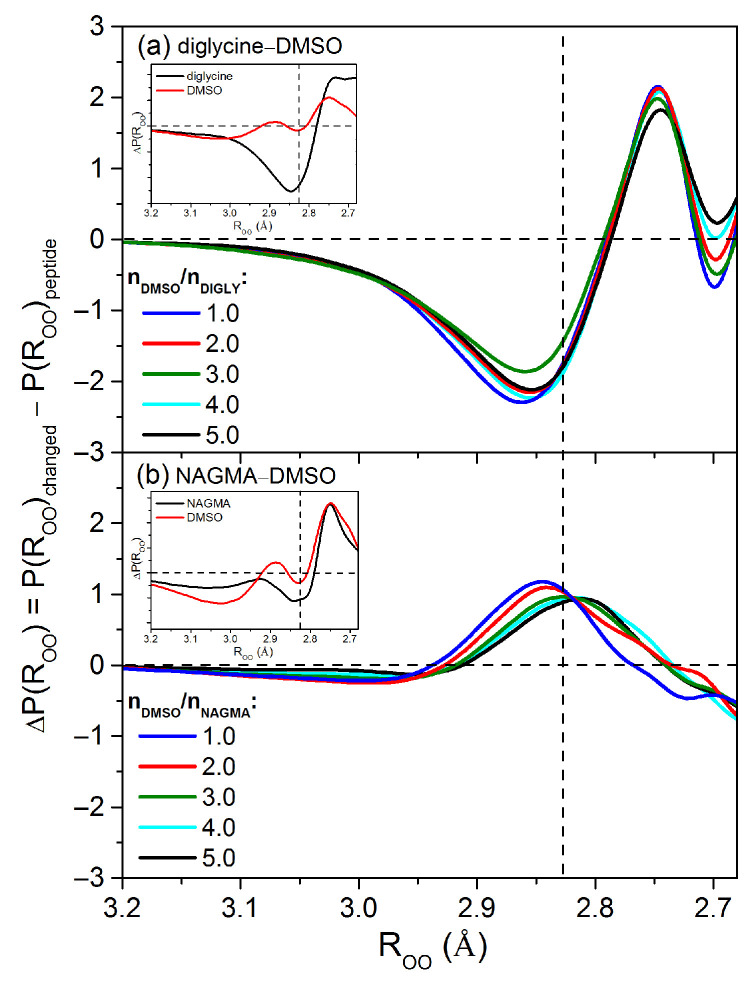
Differences in the interatomic oxygen–oxygen distance distribution function of changed-affected water, $P{\left(R_{OO}\right)}_{changed}$ (from Figure S6), and water affected by the peptide, $P{\left(R_{OO}\right)}_{peptide}$ (from Figure S5) for different molar ratios of DMSO to peptide in (**a**) diglycine–DMSO and (**b**) NAGMA–DMSO systems. Inset: Differences between interatomic oxygen–oxygen distance distribution function of water affected by pure solutes (from Figure S5), $P{\left(R_{OO}\right)}_{affected}$, and the bulk water, $P{\left(R_{OO}\right)}_{bulk}$, (from Figure S4). The vertical dashed line corresponds to the value of the most likely oxygen–oxygen distance in bulk water (2.827 Å, see Table 1).

Detailed insights into the impact of DMSO on the peptide’s hydration shell are provided by the differences in the oxygen–oxygen distance distributions between the changed-affected water and the peptide-affected water (Figure 5). The differences, $\Delta P(R_{OO})$, obtained for various molar ratios of DMSO to peptide in the diglycine–DMSO system (Figure 5a) indicate that the presence of DMSO in the peptide solution reduces the population of water molecules with mean and weak hydrogen bonds (distances equal to and longer than the most likely distance in bulk water, $R_{OO}$ ≈2.83 Å) while increasing the population of water molecules with strong hydrogen bonds (oxygen–oxygen distances of ca. 2.75 Å) compared to the water around diglycine. In this system, no effect of DMSO concentration on the peptide’s hydration shell is observed.

In the case of the NAGMA–DMSO system (Figure 5b, see also Figure S6b), we do not observe a single distinct population of water molecules as for the diglycine–DMSO system (Figure 5a, see also Figure S6a). Instead, we see an extended range of different water molecule populations, covering distances slightly longer, shorter, and the same as the most likely distance in bulk water (ca. 2.83 Å), relative to the water surrounding NAGMA. As a result, for all studied molar ratios of DMSO to peptide in the NAGMA–DMSO system, we observe a slight weakening of the hydrogen bonds between water molecules in the hydration sphere of NAGMA. Additionally, the changes in the hydration sphere of the NAGMA peptide are dependent on the concentration of DMSO.

The effect of DMSO on the peptides can be explained by referring to the hydrogen bond distributions around the pure solutes (peptides and DMSO, see Figure S5 in the Supplementary Materials), as illustrated by the differences in the oxygen–oxygen distance distributions between the solute-affected water and bulk water (inset in Figure 5). As can be seen (inset in Figure 5), in the DMSO-affected water, two populations of water molecules can be distinguished: a relatively small share of water molecules with very weak hydrogen bonds ($R_{OO}$ ≈2.9 Å) and a more pronounced share of water molecules with strong hydrogen bonds ($R_{OO}$ ≈2.75 Å). In our previous work [25], we found that the population of weakened hydrogen bonds of water molecules is located near the sulfoxide group and corresponds to water molecules improperly fitted to the water molecules hydrogen-bonded to the oxygen atom of DMSO. On the other hand, the population of strong hydrogen bonds results from the cooperation of hydrogen bonds between water molecules involved in forming a cage around the methyl groups with water molecules interacting directly with the sulfonic oxygen atom.

The hydration shell of diglycine is characterized by an increased population of strong water hydrogen bonds, compared to bulk water, significantly greater than that in the case of DMSO (see inset in Figure 5a). Therefore, it can be expected that the population of strong hydrogen bonds around DMSO interacts with the strengthened hydration shell of diglycine, leading to further reinforcement of the water structure around the peptide.

Analysis of the differences in the oxygen–oxygen distance distributions for NAGMA-affected water and DMSO-affected water in relation to bulk water (inset in Figure 5b) shows that they are very similar in terms of the magnitude of the effect, especially the enhancing effect. The comparison of the distributions of hydrogen bonds in the hydration shells of the peptide and DMSO, as well as the differences between them, are shown in Figure S5b in the Supplementary Materials. The increased population of water hydrogen bonds at distances similar to the most likely distance in bulk water, as well as those slightly longer and shorter (distance range from ca. 2.75 Å to ca. 2.92 Å), in the changed-affected water relative to the hydration sphere of NAGMA (Figure 5b), suggests that the interactions of the peptide and DMSO hydration shells involve these populations of water molecules. As a result, we observe only a very slight weakening of the hydrogen bonds between water molecules in the hydration sphere of NAGMA in the presence of DMSO.

### 2.3. Interactions of Solute Hydration Shells–DFT/ONIOM Studies

**Figure 6:**
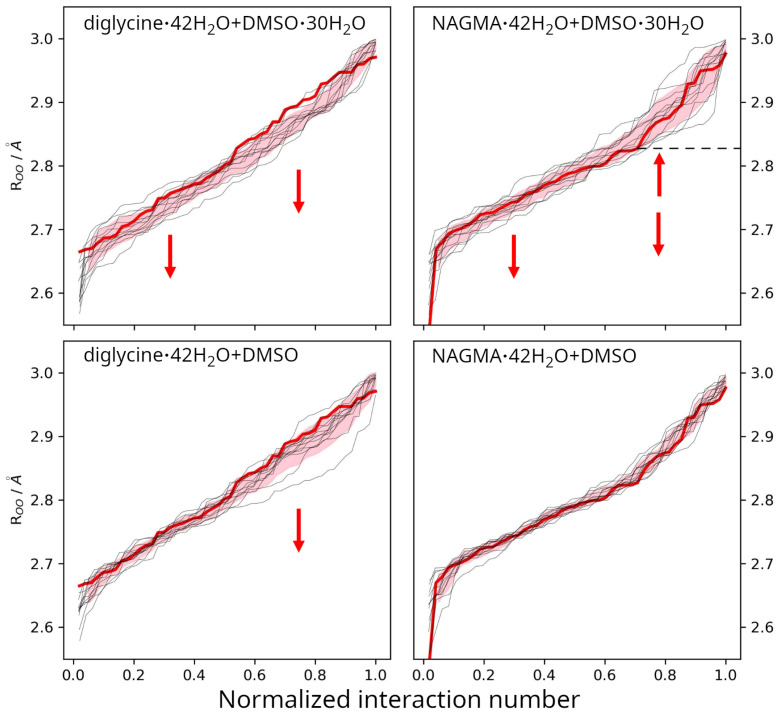
Distributions of O⋯O distances in diglycine–DMSO and NAGMA–DMSO systems with and without full DMSO hydration shells as a function of the normalized interaction number. The O⋯O interactions in each structure were sorted by length, and the assigned numbers were divided by the maximum number of interactions in the system. The interaction numbers, which are variable in supercomplexes and have different variants of the same supercomplex structure, were thus normalized to a common maximum value of 1.0 in the range of 2.55–3.0 Å for easier comparison. Red lines indicate distributions for model peptides with their full hydration shells, while black lines indicate distributions in the presence of DMSO. Optimized structures of all systems, on which the distributions are based, are presented in Supplementary Materials, Figures S7–S9. Arrows indicate the most significant changes. Red areas correspond to a single standard deviation of the mean from the distributions of systems containing DMSO. The dashed line indicates the inflection point separating stronger and weaker populations of water molecules around NAGMA.

DFT calculations allow for a better understanding or prediction of phenomena occurring in solutions by identifying specific intermolecular interactions or their absence and characterizing the hydration shell of the central solute. These aspects help validate and understand the experimental results of both ATR-FTIR and HDO FTIR studies. However, DFT/ONIOM calculations, even when repeated in different configurations, may less accurately reflect the dynamic nature of the studied systems while better representing actual intermolecular interactions. Consequently, the distributions of O⋯O distances are presented as lines rather than histograms or $R_{OO}$ distributions, as shown in Figure 6, to avoid manipulation and misinterpretation due to the small amount of data.

Direct contact was not observed between the central molecule and DMSO in any of the optimized supercomplexes (i.e., the system consisting of NAGMA or diglycine with their hydration shells and DMSO with or without its hydration shell). The overall results are consistent with the ATR-FTIR experimental data aimed at detecting changes in the vibrational structure of solutes in mixed aqueous solutions (see Section 2.1).

#### 2.3.1. Water Around Diglycine and NAGMA

The NAGMA line itself is positioned lower throughout the whole interaction number range than the line corresponding to diglycine, so it can be expected in the experiment that the O⋯O distances will be on average stronger in the case of NAGMA than diglycine, which was actually observed. A comparison of the red lines, which correspond to the pure solutes and their hydration shells, indicates that the overall spread of O⋯O distances in the case of diglycine is more uniform (the thick red line on the graph is close to a straight line) and falls within the range of approximately 2.67–2.97 Å, whereas in the case of NAGMA, the overall population spread is wider (approximately 2.55–2.98 Å), and the shape of the line deviates significantly from a straight line. The slope of such a line indicates the spread, and the smaller the slope, the smaller the spread of O⋯O distances. In the case of NAGMA, this line is “broken” around 0.8, suggesting the existence of two populations: stronger bonds with distances of 2.7–2.8 Å with a smaller spread (smaller slope), and looser bonds of 2.8–3.0 Å with a wider spread (larger slope). The inflection point is marked in Figure 6 with a dashed line. An analogous distribution of distances was observed in the experimental differences in the distance distributions between NAGMA-affected water and bulk water (inset in Figure 5b), which reveals an increased population of the strong hydrogen bonds (positive $\Delta P(R_{OO})$ values) and a slightly decreased population of the weak hydrogen bonds (negative $\Delta P(R_{OO})$ values) relative to bulk water. In the case of diglycine, a similar division into populations of stronger and weaker interactions cannot be observed.

#### 2.3.2. Water in Diglycine—DMSO Systems

In Figure 6, the distribution of O⋯O distances for the hydration complex of diglycine is marked with a bold red line, and other colors represent the O⋯O distributions around diglycine in its supercomplexes with DMSO and its hydration shell. Comparing the curves shows that the presence of DMSO shortens the distances in diglycine’s hydration shell, as evidenced by the downward shift of the supercomplex curves toward shorter O⋯O distances (the red-filled area under the red line, emphasized with arrows). This indicates that stronger hydrogen bonds, even below 2.6 Å, and medium-range distances (around 2.8 to 2.95 Å) are present in the supercomplexes. The enhancing effect of DMSO on the hydrogen bonds of water in the hydration shell of diglycine is consistent with experimental results (see Section 2.2.3). In some supercomplexes, the population of strong interactions weakens (curves above the red line in the range of approximately 2.7 to 2.8 Å), but no single cause was identified for this phenomenon. All supercomplexes also show a small population of weaker (longer) O⋯O interactions compared to the initial complex (above 2.95 Å).

A similar distribution of O⋯O distances is observed for the supercomplex where a single DMSO molecule approaches diglycine with its full hydration shell. Qualitatively, the changes are the same, but the magnitude is smaller, especially in the lower $R_{OO}$ region. The observed changes in both cases suggest that the presence of DMSO triggers a kind of “defensive” response from diglycine’s hydration shell, which strengthens itself to prevent direct interactions between diglycine and DMSO.

#### 2.3.3. Water in NAGMA–DMSO Systems

The situation in the NAGMA–DMSO system is different from the previous case. There is no clear tendency for NAGMA’s hydration shell to strengthen or weaken in the presence of DMSO and its shell, as in the case of diglycine. The systems react across the entire range of distances, with some distribution lines above and some below the reference line for the NAGMA·42H_2_O complex. This is also confirmed by experimental differences in the distribution of distances between changed-affected water and water around NAGMA (Figure 5b), where the change caused by the presence of DMSO, although slightly shifted towards shorter H-bonds, just adds to the populations of somewhat weaker and stronger interactions. This lack of clear changes in the population of O⋯O interactions in NAGMA’s shell is observed not only in the supercomplex with a fully hydrated DMSO but also in the case of the supercomplex with free DMSO. Moreover, in the case of the latter, the hydration shell of NAGMA holds up regardless of the calculated structure. This remains consistent with our earlier results regarding the hydration of both peptides, which indicated significant differences in both cases [11], and the NAGMA shell itself was found to have a small number of potential interaction sites. This suggests that DMSO has a more neutral effect on the compact hydration shell of this dipeptide compared to diglycine, or even no effect at all in the case of the system that simulates a more concentrated NAGMA and DMSO solution.

## 3. Materials and Methods

### 3.1. Chemicals and Solutions

*N*-acetyl-glycine-methylamide (NAGMA, 2-acetamido-*N*-methylacetamide) (prepared according to the procedure described in ref. [11]), diglycine (≥99%, Aldrich, Darmstadt, Germany), dimethyl sulfoxide (99.9+%, Alfa Aesar, Kandel, Germany) and deuterium oxide (isotopic purity 99.96%, Aldrich, Darmstadt, Germany), were used as supplied in experiments concerning FTIR hydration water investigation. Deionized water κ<0.01 S·cm^–1^ was used for the preparation of all solutions in the case of hydration experiments. All solutions, in both kinds of experiments, were prepared by weight, and their densities were measured by means of the Anton Paar DMA 5000 densimeter (Graz, Austria) at 25.000 ± 0.001 °C.

The pH of aqueous peptide solutions was measured using a Handylab pH 11 pH-meter (SHOTT Instruments GmbH, Mainz, Germany). The pH of aqueous peptide solutions in the concentration range of 0.1–0.4 $\mathrm{mol}\;\cdot \;{\mathrm{dm}}^{-3}$ was 5.9 ± 0.1 for diglycine and 6.5 ± 0.1 for NAGMA.

### 3.2. ATR-FTIR Studies of Weak Peptide–DMSO Interactions

A variant of the difference spectra method was used to extract information on possible interactions and their type in solutions of diglycine–DMSO and NAGMA–DMSO, similar to the method presented in refs. [26,27,28]. In the first stage, a series of dilutions of diglycine, NAGMA, and DMSO in water were prepared in the range from 0.05 to 0.40 $\mathrm{mol}\;\cdot \;{\mathrm{dm}}^{-3}$. There were six ATR-FTIR spectra in each series. They were all taken at 25 °C with 256 scans and a resolution of 1 ${\mathrm{cm}}^{-1}$ using the single-reflection diamond crystal ATR attachment for the FTIR spectrometer Invenio-R (Bruker, Rosenheim, Germany). We measured several water vapor spectra over the course of the experiments and later subtracted them from each of the proper spectra using a vapor subtraction algorithm [29]. From each spectrum, the spectrum of pure water was subtracted with a subtraction coefficient equal to $C_{H_{2}O}/C_{H_{2}O}^{o}$, where the molar concentration of pure water $C_{H_{2}O}^{o}=55.33$ $\mathrm{mol}\;\cdot \;{\mathrm{dm}}^{-3}$, and $C_{H_{2}O}$ was the current concentration of water in the sample. The spectra were finally divided by the molar concentration of the solute, thus obtaining three series of molar spectra for each solute. From each molar spectrum in the series, except for the spectrum corresponding to the lowest concentration, the spectrum corresponding to the lowest concentration was subtracted with a coefficient of 1. This resulted in a series of difference spectra, which were averaged to obtain the average difference spectrum in the series. This spectrum indicates the average changes in the positions of the solute bands as a result of concentration changes.

Similarly, a series of spectra of the ternary mixtures diglycine–water–DMSO and NAGMA–water–DMSO were prepared and measured. The concentration of DMSO in each of these series was constant and equal to 0.40 $\mathrm{mol}\;\cdot \;{\mathrm{dm}}^{-3}$, while the concentration of diglycine or NAGMA varied in the range from 0.05 to 0.40 $\mathrm{mol}\;\cdot \;{\mathrm{dm}}^{-3}$. From such spectra, the spectrum of pure water was also subtracted with the appropriate coefficient resulting from its molar concentration in the sample and with the spectrum of DMSO corresponding to the same concentration as in each of the samples. In this way, the spectra of diglycine or NAGMA in the ternary systems were isolated, which were then divided by the molar concentration of these solutes in each sample to obtain their molar spectra. As with the pure solutes, difference spectra were obtained by subtracting the molar spectrum corresponding to the lowest concentration from each subsequent spectrum in the series with a coefficient of 1. These spectra were finally averaged. As before, the average spectrum indicates the average changes in the positions of the solute bands as a result of concentration changes, but this time in the presence of DMSO. Any differences between these average difference spectra and the average difference spectra in the diglycin–water or NAGMA–water systems will indicate or negate the possibility of forming relatively strong and direct interactions in the ternary systems due to the presence of DMSO.

All spectra were recorded and analyzed using OPUS 8.7 (Bruker, Rosenheim, Germany) and Python 3.7 scripts developed for the complex spectra analysis.

### 3.3. FTIR Investigation of Water Structure–HDO Difference Spectra Method

#### 3.3.1. Sample Preparations

Peptide–water systems: Stock solutions of peptides (diglycine or NAGMA), i.e., in the two-component system (peptide–water), with a molality of approximately 0.1 $\mathrm{mol}\;\cdot \;{\mathrm{kg}}^{-1}$ were obtained by dissolving the peptide in deionized water. Subsequently, an appropriate amount of this solution was weighed and divided into two parts to prepare a sample and reference solution. The sample solution was prepared by adding D_2_O to one of the parts in a weight ratio of 4% relative to the amount of H_2_O in the sample. The reference solution was obtained by the addition of an equivalent amount of H_2_O to the second part of the solution. It was confirmed that this amount of deuterium oxide is sufficient for the reaction $H_{2}O+D_{2}O=2\mathrm{HDO}$ (equilibrium constant *K* ≈ 4) to achieve an almost quantitative conversion of HDO [30]. The amount of D_2_O added to H_2_O allowed, in accordance with the mentioned equilibrium constant indicated in ref [30], for the quantitative conversion of D_2_O to HDO with a final HDO concentration that permits the registration of HDO bands without saturating the detector, i.e., with an absorbance below 1 at the assumed IR cell thickness.

Peptide–DSMO–water systems: Aqueous solutions of ternary systems (peptide–DMSO–water) were obtained by weighing the appropriate amount of the peptide stock solution and adding various amounts of DMSO. The solutions were then divided into two parts and processed in a manner similar to the process used for aqueous peptide solutions. In this way, a series of solutions (reference and sample) with a constant peptide concentration (approximately 0.1 $\mathrm{mol}\;\cdot \;{\mathrm{kg}}^{-1}$) and varying DMSO concentrations (approximately 0.1, 0.2, 0.3, 0.4, and 0.5 $\mathrm{mol}\;\cdot \;{\mathrm{kg}}^{-1}$) were prepared.

DMSO–water systems: The DMSO stock solution in water, i.e., in the two-component system (DMSO–water) with a molality of approximately 0.5 $\mathrm{mol}\;\cdot \;{\mathrm{kg}}^{-1}$ was prepared by dissolving DMSO in deionized water. Subsequent solutions with progressively decreasing molality were obtained by diluting the stock solution of DMSO with an appropriate amount of deionized water. To obtain a series of semi-heavy water samples, each solution was divided into two parts. The further procedure was analogous to the preparation of aqueous peptide solutions. As a result, a series of DMSO water solutions (reference and sample) with molalities of approximately 0.1, 0.2, 0.3, 0.4, and 0.5 $\mathrm{mol}\;\cdot \;{\mathrm{kg}}^{-1}$ were obtained.

#### 3.3.2. FTIR Measurements

FTIR experiment: FTIR spectra of prepared solutions were recorded on the Invenio-R FTIR spectrometer (Bruker, Rosenheim, Germany) (500–5000 cm^–1^, resolution of 4 cm^–1^, 128 scans for each spectrum). A liquid transmission cell (model A145, Bruker Optics, Ettlingen, Germany) with CaF_2_ windows separated with PTFE spacers was used. The path length was determined interferometrically (28.84 μm). The temperature of measurements was kept at 25 ± 0.1 °C and monitored using an electronic thermometer with thermocouples inserted into the cell. The spectrometer’s interior was purged with dry nitrogen, produced by the generator (Claind Brezza NiGen LCMS 40-1, Lenno, Italy), to eliminate the influence of air components on a recorded spectra shape, namely, water vapor and carbon dioxide.

All spectra were recorded and analyzed using the commercial PC software: OPUS 8.7 (Bruker, Rosenheim, Germany), GRAMS/AI version 9.3 (Thermo Fisher Scientific Inc., Waltham, MA, USA), RazorTools/8 (Spectrum Square Associates, Inc., Ithaca, NY, USA, run under GRAMS/AI) and Python 3.7 scripts developed for the complex spectra analysis.

#### 3.3.3. Analysis of HDO Spectral Data

The difference spectra method was used to extract the solute-affected HDO spectra for various molalities of aqueous solutions, i.e., the spectra of the water altered by interactions with the solute. This method is based on the assumption that water in the solution can be divided into two additive parts: “affected” water (i.e., water changed by interactions with the solute) and “unaffected” water (“bulk” water, i.e., water displaying the characteristics of pure water). The spectrum of affected water at molality *m* can be calculated by means of the following Equation (2):

(2) $$\varepsilon_{a}=\frac{1}{NMm}\left(\varepsilon -\varepsilon_{b}\right)+\varepsilon_{b}$$

where $\varepsilon_{a}$ and $\varepsilon_{b}$ are, accordingly, the molar absorption coefficients of affected water and bulk water; ε is the molar absorption coefficient of water in solution at the molality of the solute *m* $\mathrm{mol}\;\cdot \;{\mathrm{kg}}^{-1}$); the parameter *N*, called the affected number, is the number of moles of water affected by one mole of solute; and *M* is the mean molar mass of water (including mass of D_2_O in HDO spectra) ($\mathrm{kg}\;\cdot \;{\mathrm{mol}}^{-1}$). The method was described in detail in refs. [31,32,33] and also in the Supplementary Materials of ref. [12].

The solute-affected water spectra in systems containing two solutes (solute A–solute B–water) were obtained with respect to the molality of the solute in excess (according to Equation (2)). Detailed information on the analysis of affected water spectra in ternary systems can be found in ref. [24]. The idea is simple: according to the procedure described above, we obtain the spectra of HDO affected by the pure components A and B (in this article, DMSO and peptide) and simultaneously obtain the corresponding numbers of affected water molecules (*N*). Similarly, we obtain the spectrum of affected water in the system composed of A and B (A-B) along with the number *N*. Ideally, if both A and B act completely independently in solution, the A-B affected spectrum should be the sum of the pure affected spectra of A and B. However, the situation becomes non-ideal when any interactions occur. In such cases, the actual A-B affected spectrum differs from the ideal A-B affected spectrum. Evaluating the contribution of each pure affected spectrum of A or B to this non-ideal deviation is challenging. Therefore, in ref. [24], we proposed an algorithm based on the Monte Carlo method to analyze these deviations. This algorithm seeks the best fit of the pure A and B affected spectra to the A-B affected spectrum—a non-trivial task. The parameters of this fitting provide insights into the contributions of the pure A and pure B affected spectra to the A-B affected spectrum and corresponding *N* numbers, as well as the spectrum and contribution of the non-ideal component. For HDO spectra, this additional non-ideal contribution is interpreted as the spectrum of HDO water molecules affected by interactions between both solutes.

The shapes of the spectral bands were transformed into interatomic oxygen–oxygen distance distribution functions, $P(R_{OO})$, using an empirical function [34]:

(3) $$P\left(R_{OO}\right)=C\varepsilon \left(\nu \right)\left(d\nu /dR_{OO}\right)\;,$$

where *C* is a normalization constant and $\left(d\nu /dR_{OO}\right)$ is obtained by analytically differentiating the relation from Equation (4):

(4) $$\nu_{\mathrm{OD}}\;/\;{\mathrm{cm}}^{-1}=2727-\exp\left[16.01-3.73\left(R_{OO}\;/\;Å\right)\right]\;.$$

This correlation was derived from the positions of HDO bands in solid hydrates measured by infrared spectroscopy and the corresponding intermolecular distances determined by diffraction methods [34].

Details concerning the extraction, the interpretation of the solute-affected water spectrum, as well as the transformation of HDO spectral contours into the probability distribution of the intermolecular oxygen–oxygen distance, $P(R_{OO})$, have been described in refs. [31,32,33] and also in the Supplementary Materials of ref. [12].

### 3.4. DFT/ONIOM Calculations

To simulate the effect of DMSO on the hydration shells of diglycine and NAGMA, theoretical DFT calculations (M06-2X functional [35]) were used, along with the ONIOM approach (Our own N-layered Integrated Molecular Orbital and Molecular Mechanics [36]), which combines more complex quantum calculations for key parts of the system with simpler models for the rest to efficiently study large chemical systems.

The hydration shells for all solutes were prepared following a previously described procedure [25]. In short, this method involves gradually adding water molecules to the central molecule (diglycine, NAGMA, or DMSO) one by one in the initial steps (up to 6 water molecules) and later in groups of 2–5, in positions that ensure the lowest possible energy for the entire hydration complex after the optimization step. The central molecule is calculated at the higher level of theory (M06-2X/aug-cc-pVTZ) and the hydration layer at the lower one (M06-2X/cc-pVDZ). This way, closed hydration shells were obtained for DMSO (30 water molecules) and NAGMA (42 water molecules). For diglycine, which exists as a zwitterion in aqueous solution (containing both positive and negative charges), it was necessary to initially stabilize the free NH_3_^+^ and COO^–^ groups by optimizing a starting complex where three water molecules surrounded each of these functional groups. This stabilized structure prevented the “proton hopping” between these groups, which would otherwise neutralize their charges. Once the optimal starting complex for diglycine·6H_2_O was obtained in this form, the calculations were continued as described above for up to 42 water molecules.

The next step involved creating supercomplexes, which are systems composed of fully hydrated NAGMA or diglycine complexes and DMSO, either with or without its hydration shell. This resulted in four types of systems: NAGMA·42H_2_O + DMSO·30H_2_O, NAGMA·42H_2_O + DMSO, diglycine·42H_2_O + DMSO·30H_2_O, diglycine·42H_2_O + DMSO. Supercomplexes with hydrated DMSO simulated more diluted solutions, while those with free DMSO molecules represented more concentrated solutions. In each case, DMSO was attached to the central NAGMA or diglycine complex (with or without its hydration shell) in 12 different configurations to explore a wide variety of possible interactions. The geometries of supercomplexes were then optimized in the same way as the smaller hydration complexes. None of the optimized structures exhibited negative frequencies.

For each hydration complex and supercomplex, O⋯O distances in the layer up to 3.5 Å from the central molecule were calculated. This distance corresponds to the average thickness of the first hydration layer. All possible O⋯O distances in this layer were calculated and then restricted to the range of 0.255–3.00 Å, covering distances characteristic of hydrogen bonds from strong to weak. Since the number of O⋯O interactions could vary depending on the central molecule, as well as between structures for the same molecule and its hydration shell, the total number of interactions within the given range of 0.255–3.00 Å was normalized to a value of 1. This approach facilitated the comparison of calculation results between structures and central molecules.

Although the preparation of 12 configurations for each of the supercomplex structures provided a good variety, DFT still offers a rather static picture of the situation in the solution compared to methods like molecular dynamics simulations. However, we focused on accurately reflecting the real intermolecular interactions in the hydration layers, accounting for electronic effects and structural changes in the water molecules.

All starting structures were prepared using the Avogadro program [37], and the calculations were performed using the Gaussian 2016 software package [38]. Computations were carried out using the computers of the Centre of Informatics Tricity Academic Supercomputer & Network.

## 4. Conclusions

In this paper, the interactions of dimethyl sulfoxide (DMSO) with two small peptides, diglycine and *N*-acetyl-glycine-methylamide (NAGMA), in aqueous solutions were investigated using FTIR spectroscopy and computational methods.

ATR-FTIR spectroscopy revealed no significant shifts in the vibrational frequencies of the S=O bonds in DMSO or the N–H and C=O bonds in the peptides, indicating a lack of direct interactions between DMSO and the peptides. DFT calculations further confirmed the absence of direct contact between the peptides and DMSO in the optimized systems. This suggests that DMSO indirectly affects both peptides by modifying the structure of water around them.

The use of FTIR spectroscopy, supported by the HDO difference spectra method, provided insight into the structural and energetic characteristics of the hydration shells of the peptides and DMSO, as well as the interactions between these shells. Analysis of HDO spectral data allowed the isolation of the contribution of water molecules influenced by both DMSO and the peptides, elucidating how the hydration shells of the peptides were modified in the presence of DMSO. The results reveal that the contact between the hydration shells of the peptides and DMSO is minimal and occurs at the surface of their shells. Notably, the hydrogen bond network around DMSO remains intact due to interactions with the hydration shells of both peptides. However, interactions between DMSO and the peptides lead to changes in the hydration shells of the peptides, but the nature of these interactions varies.

In the diglycine–DMSO system, the presence of DMSO increases the hydrogen bond energy between the water molecules surrounding diglycine. This effect is attributed to the interactions of water molecules with strong hydrogen bonds around DMSO with the reinforced hydrogen bond network in the hydration sphere of diglycine, resulting in an additional strengthening of diglycine’s hydration shell. In contrast, DMSO exerts a less pronounced effect on the NAGMA peptide, likely due to the similarities between the hydration spheres of NAGMA and DMSO. The slight weakening of hydrogen bonds in the hydration shell of NAGMA is caused by the interactions of water molecules with medium hydrogen bond energies in the hydration spheres of both solutes, leading to a dilution of NAGMA’s hydration sphere.

Theoretical results obtained from DFT/ONIOM calculations supported these experimental observations. In diglycine–DMSO supercomplexes, the presence of DMSO leads to shorter O⋯O distances in the peptide’s hydration shell, indicating stronger hydrogen bonds. However, no consistent trend was observed in the NAGMA–DMSO supercomplexes regarding the strengthening or weakening of the hydration shell.

These findings suggest that DMSO can influence the stability of peptides in different ways depending on the structural features of the peptides, which, in turn, shape the properties of their hydration shells. Overall, this study provides valuable insights into the interactions between small peptides and DMSO, highlighting the importance of hydration shells in mediating these interactions. The findings underscore the complexity of protein-solvent interactions and the need for further research to fully understand the mechanisms by which DMSO and other small organic co-solutes affect protein stability and functionality.

## Figures and Tables

**Table 1:** The most important parameters of the HDO band of the diglycine-affected water, NAGMA-affected water, DMSO-affected water, bulk water, and the changed-affected water for different molar ratios of DMSO to peptide, and the respective intermolecular oxygen–oxygen distances. $R_{OO}$ errors have been estimated on the basis of the HDO band’s position errors. Other parameters of HDO bands are presented in Tables S1 and S2 in Supplementary Materials. ^*a*^ The molar ratio of DMSO to peptide (approximate values). ^*b*^ Band position at maximum (cm^−1^). ^*c*^ Band position at the gravity center (cm^−1^). ^*d*^ The most likely O⋯O distance (Å). ^*e*^ Mean O⋯O distance (Å).

$n_{\mathrm{DMSO}}$/$n_{\mathrm{peptide}}$ ^*a*^	$\nu_{\mathrm{OD}}^{o}$ ^*b*^	$\nu_{\mathrm{OD}}^{g}$ ^*c*^	$R_{\mathrm{OO}}^{o}$ ^*d*^	$R_{\mathrm{OO}}^{g}$ ^*e*^
Pure Solutes				
diglycine	2496 ± 2	2449 ± 2	2.778 ± 0.003	2.802 ± 0.003
NAGMA	2505 ± 2	2488 ± 2	2.827 ± 0.003	2.834 ± 0.003
DMSO	2514 ± 2	2483 ± 2	2.821 ± 0.003	2.831 ± 0.003
bulk water	2509 ± 2	2497 ± 2	2.827 ± 0.003	2.844 ± 0.003
Changed-Affected Water in Diglycine–DMSO System				
1.0	2415 ± 2	2349 ± 2	2.744 ± 0.003	2.729 ± 0.003
2.0	2415 ± 2	2356 ± 2	2.744 ± 0.003	2.732 ± 0.003
3.0	2419 ± 2	2363 ± 2	2.744 ± 0.003	2.739 ± 0.003
4.0	2417 ± 2	2354 ± 2	2.744 ± 0.003	2.729 ± 0.003
5.0	2417 ± 2	2361 ± 2	2.744 ± 0.003	2.732 ± 0.003
Changed-Affected Water in NAGMA–DMSO System				
1.0	2511 ± 2	2496 ± 2	2.836 ± 0.003	2.839 ± 0.003
2.0	2507 ± 2	2493 ± 2	2.836 ± 0.003	2.836 ± 0.003
3.0	2505 ± 2	2493 ± 2	2.827 ± 0.003	2.834 ± 0.003
4.0	2504 ± 2	2494 ± 2	2.822 ± 0.003	2.834 ± 0.003
5.0	2502 ± 2	2495 ± 2	2.819 ± 0.003	2.836 ± 0.003

## Data Availability

All the obtained information is available per request.

## Supplementary Materials

The following supporting information can be downloaded at: https://www.mdpi.com/article/10.3390/molecules1010000/s1. The Supplementary Materials contain the results of both ATR-FTIR and FTIR spectroscopic experiments and the structures of the hydrated complexes optimized using the DFT/ONIOM method.

## Abbreviations

The following abbreviations are used in this manuscript:

ATR	Attenuated Total Reflectance
DFT	Density Functional Theory
DMSO	dimethyl sulfoxide
FTIR	Fourier transform infrared
*fwhh*	Full width at the half-height
NAGMA	*N*-acetyl-glycine-methylamide
NALMA	*N*-acetyl-leucine-methylamide
NMA	*N*-methylacetamide
ONIOM	Our own N-layered Integrated Molecular Orbital and Molecular Mechanics
