# Supplementary material for: Characterizing Interactions Between Small Peptides and Dimethyl Sulfoxide Using Infrared Spectroscopy and Computational Methods
Source: Molecules. 2024 Dec 12;29(24):5869. doi: 10.3390/molecules29245869 (PMC11677926; doi:10.3390/molecules29245869)
Supplement: Supplementary file 1 [file molecules-29-05869-s001.zip › molecules-3310248-supplementary.pdf]

Supplementary Materials

Characterizing Interactions Between Small Peptides and Dimethyl Sulfoxide Using Infrared Spectroscopy and Computational Methods

Aneta Panuszko,* Przemysław Pastwa, Jacek Gajewski and Piotr Bruździak

Department of Physical Chemistry, Chemical Faculty, Gdańsk University of Technology

Narutowicza 11/12, 80-233 Gdansk, Poland

* *Corresponding Author E-mail:* aneta.panuszko@pg.edu.pl

S1. ATR-FTIR experiment

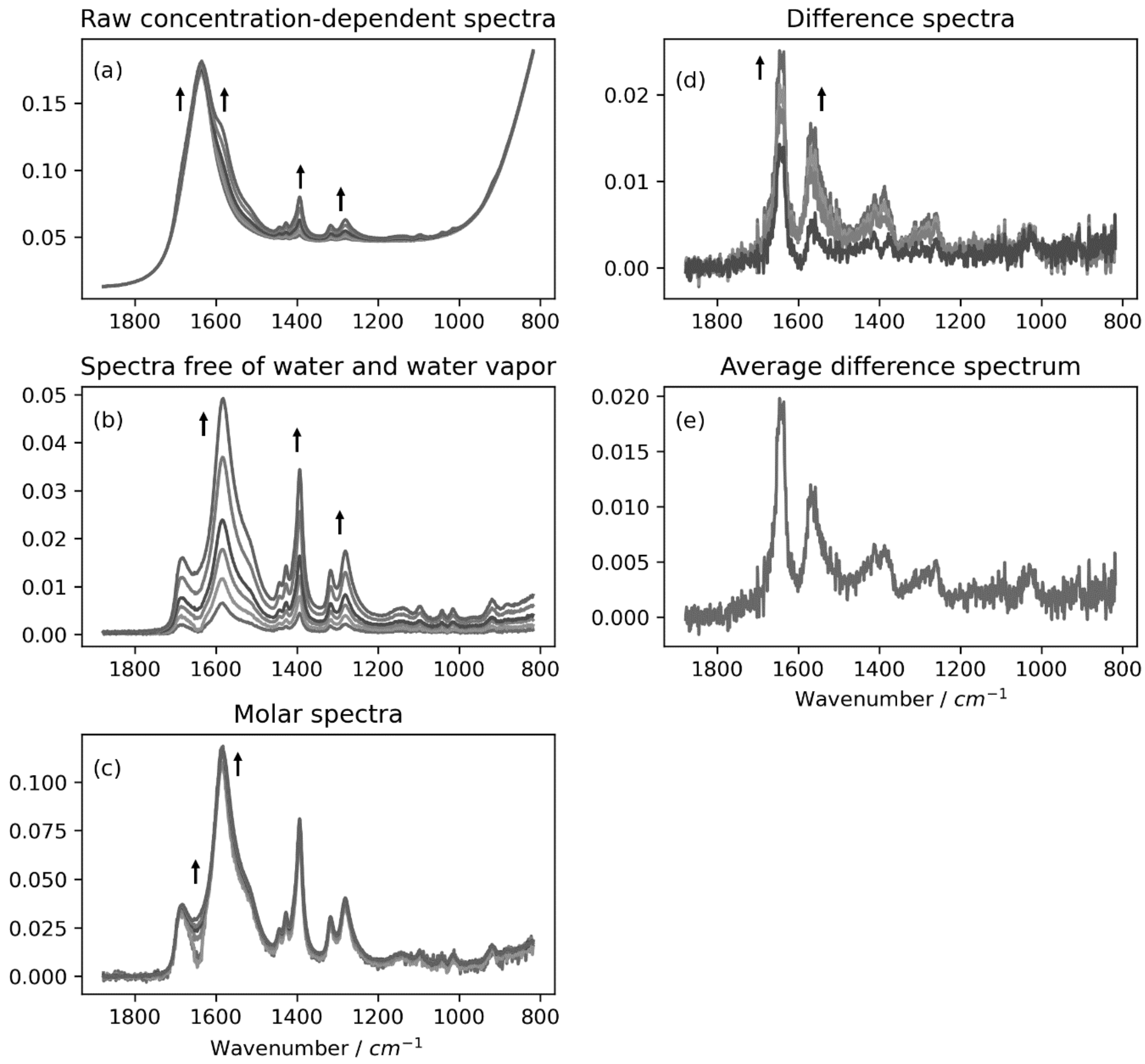

Figure S1. Method of obtaining differential spectra indicating the influence of factors (concentration, presence of DMSO) on the shape of ATR-FTIR spectra of peptides in series: **(a)** raw spectra of aqueous solutions of diglycine in the concentration range of 0.0 – 0.4 mol·dm^{-3}; **(b)** the same spectra without the contribution of water vapor and spectra of pure water; **(c)** spectra after subtraction divided by the molar concentrations of diglycine in each sample; **(d)** differential spectra resulting from the subtraction of the molar spectrum of diglycine corresponding to the lowest concentration from each remaining spectrum at higher concentrations; **(e)** average differential spectrum, resulting from averaging the differential spectra across the entire series. On the vertical axes, absorbance is marked, and on the horizontal axes, the wavenumber is indicated. The arrows indicate the direction of changes with increasing concentration.

S2. FTIR spectroscopy results

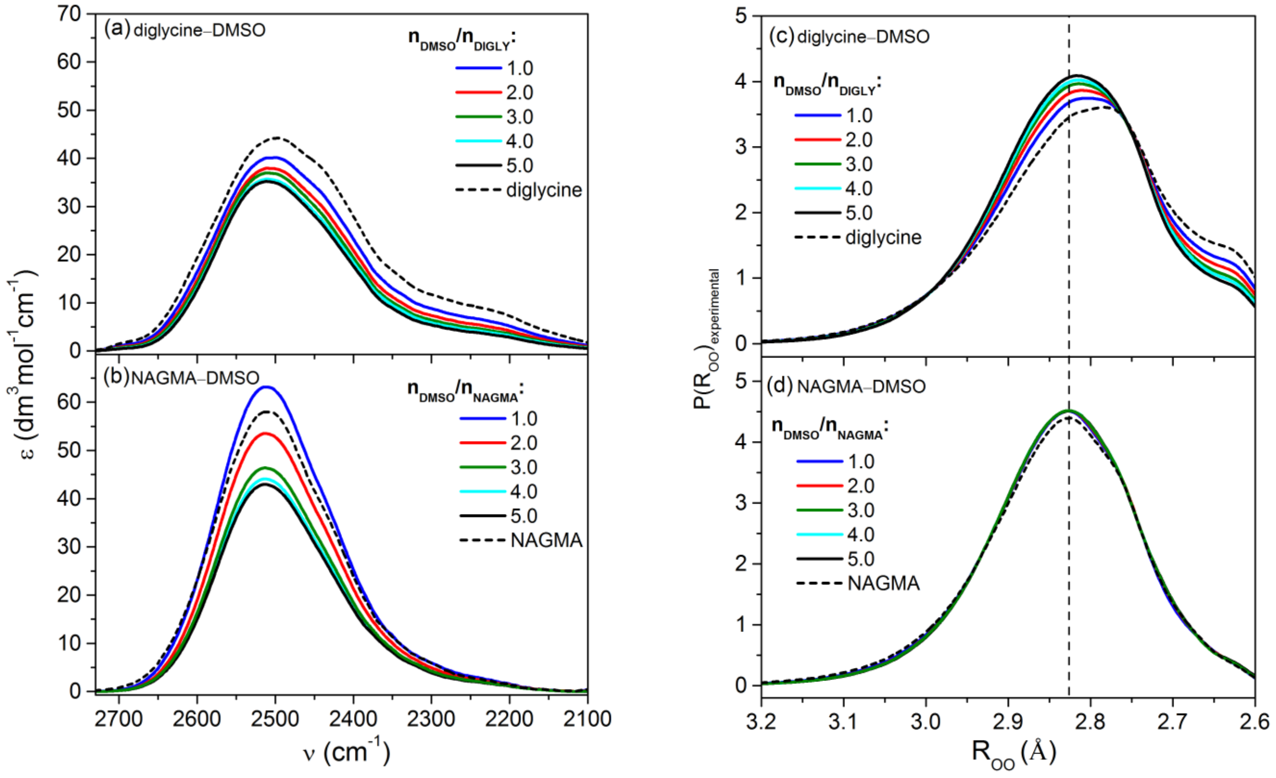

Figure S2. (a, b) Spectra of water affected by peptide and DMSO in ternary mixtures (experimental affected spectra) for different molar ratios of DMSO to peptide in (a) diglycine–DMSO and (b) NAGMA–DMSO systems, together with the peptide-affected HDO spectrum. **(c, d)** Interatomic oxygen–oxygen distance distributions function obtained on the basis of the experimental affected spectra (shown in Figs. S2a and b) for (c) diglycine–DMSO and (d) NAGMA–DMSO systems. The vertical dashed line corresponds to the value of the most likely oxygen–oxygen distance in bulk water (2.827 Å, see Table S1).

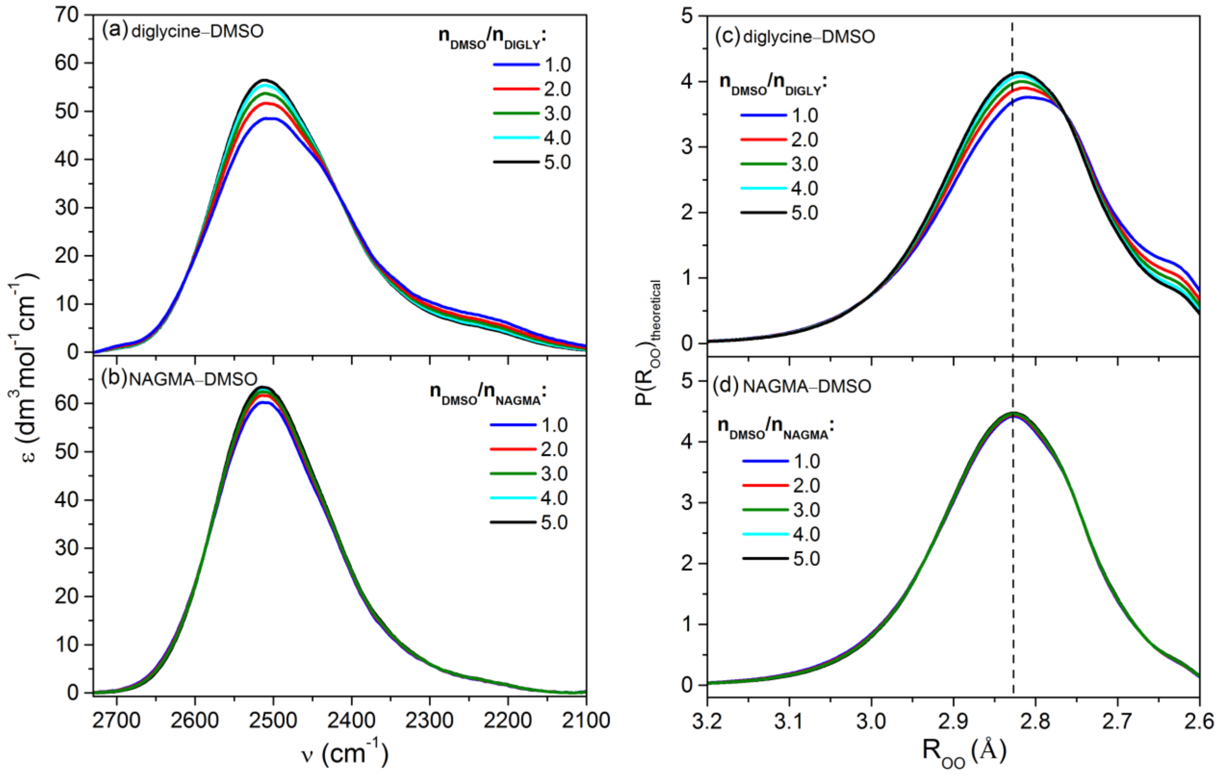

Figure S3. (a, b) Theoretical affected spectra, in the OD stretching region, for different molar ratios of DMSO to peptide in (a) diglycine–DMSO and (b) NAGMA–DMSO systems. **(c, d)** Interatomic oxygen–oxygen distance distributions function obtained on the basis of theoretical affected spectra (shown in Figs. S3a and b) for different molar ratios of DMSO to peptide in (c) diglycine–DMSO and (d) NAGMA–DMSO systems. The vertical dashed line corresponds to the value of the most likely oxygen–oxygen distance in bulk water (2.827 Å, see Table S1).

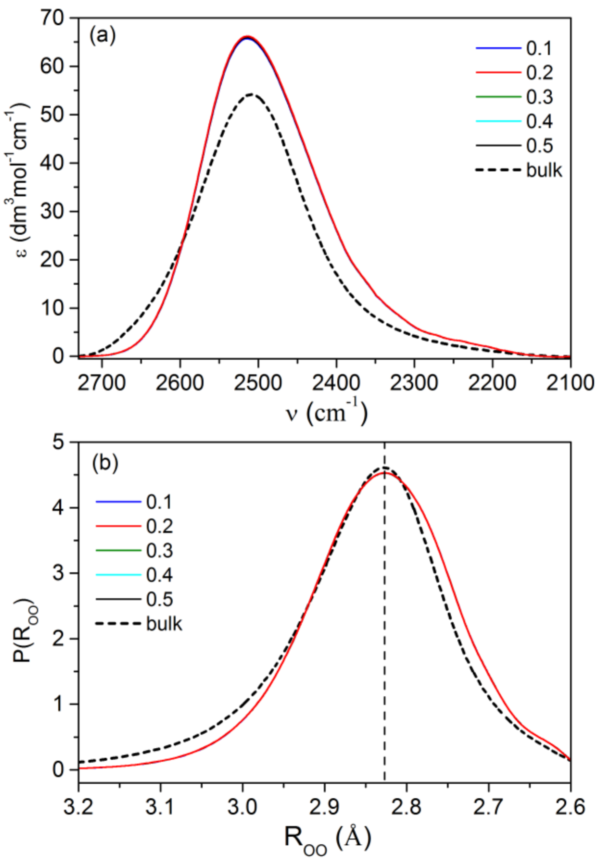

Figure S4. (a) Spectra of water affected by DMSO in the OD stretching region as a function of DMSO molality (mol·kg^{-1}), together with the bulk HDO spectrum. The spectra overlap each other. **(b)** Interatomic oxygen–oxygen distance distributions function obtained on the basis of the affected spectra (shown in Fig. S4a). The distributions overlap one another. The vertical dashed line corresponds to the value of the most likely oxygen–oxygen distance in bulk water (2.827 Å, see Table S1).

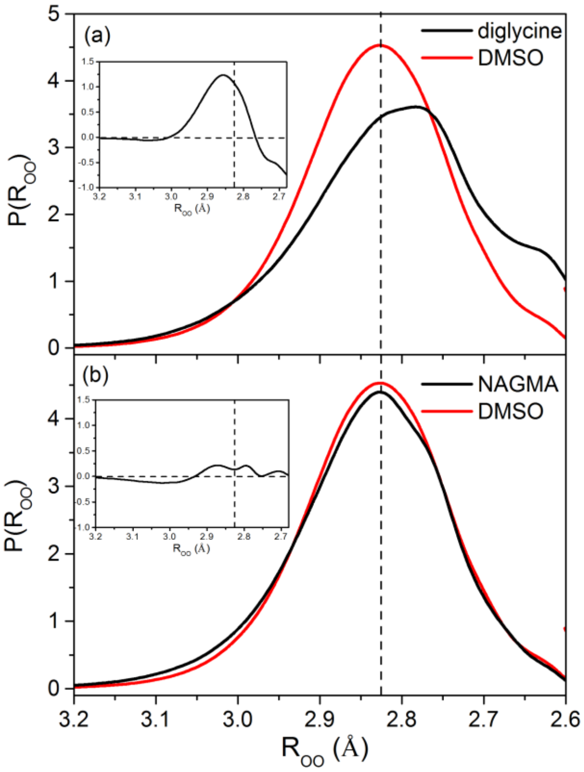

Figure S5. Comparison of oxygen-oxygen interatomic distance distribution functions for water affected by pure solutes: **(a)** diglycine (from Fig. S2c) vs. DMSO (from Fig. S4b). **(b)** NAGMA (from Fig. S2d) vs. DMSO (from Fig. S4b). Inset: Differences in the interatomic oxygen–oxygen distance distribution function of DMSO-affected water and peptide-affected water.

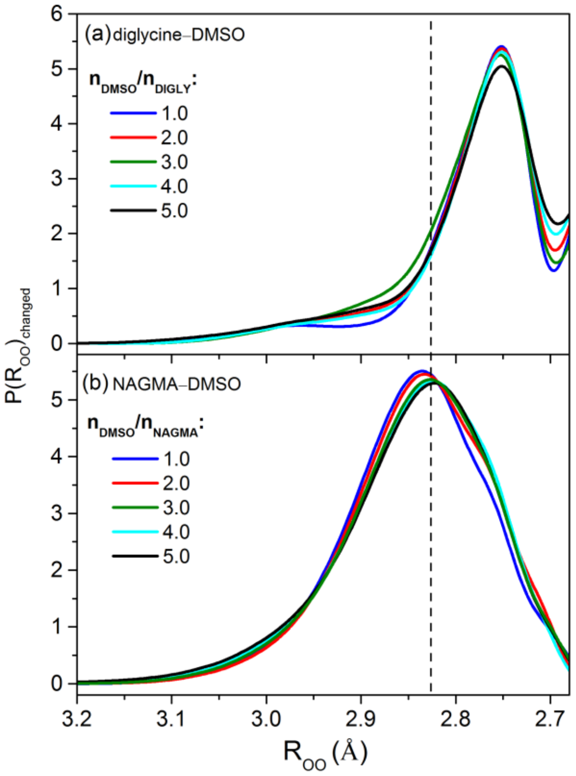

Figure S6. Interatomic oxygen–oxygen distance distributions function for different molar ratios of DMSO to peptide in **(a)** diglycine–DMSO and **(b)** NAGMA–DMSO systems obtained on the basis of the changed-affected water spectra shown in Fig. 3 and Fig. 4, respectively. The vertical dashed line corresponds to the value of the most likely oxygen–oxygen distance in bulk water (2.827 Å, see Table S1).

Table S1. The parameters of HDO band of diglycine-affected water (m_{digly} ~ 0.1 mol·kg^{-1}), DMSO-affected water, experimental and theoretical affected water and changed-affected water in diglycine–DMSO systems, the bulk water, and the respective intermolecular oxygen–oxygen distances. R_{OO} errors have been estimated on the basis of the HDO bands position errors.

m_{DMSO} [a]	N [b]	ν^{p}_{OD} [c]	ν^{g}_{OD} [d]	*fwhh* [e]	I [f]	R^{o}_{OO} [g]	R^{g}_{OO} [h]
bulk water							
	–	2509±2	2497±2	162±4	9953	2.827±0.003	2.844±0.003
diglycine							
	11.3±0.5	2496±2	2449±2	213±4	11243	2.778±0.003	2.802±0.003
DMSO							
0.1	3.0±0.5	2514±2	2483±2	169±4	12161	2.821±0.003	2.831±0.003
0.2	3.0±0.5	2515±2	2483±2	169±4	12252	2.821±0.003	2.831±0.003
0.3	3.0±0.5	2515±2	2483±2	169±4	12222	2.821±0.003	2.831±0.003
0.4	3.0±0.5	2515±2	2483±2	169±4	12212	2.821±0.003	2.831±0.003
0.5	3.0±0.5	2515±2	2483±2	169±4	12202	2.821±0.003	2.831±0.003
experimental-affected water							
0.1	16.2±0.5	2500±2	2456±2	204±4	9526	2.802±0.003	2.810±0.003
0.2	10.5±0.5	2509±2	2460±2	198±4	8627	2.815±0.003	2.815±0.003
0.3	8.8±0.5	2509±2	2463±2	191±4	8113	2.815±0.003	2.817±0.003
0.4	7.7±0.5	2511±2	2465±2	190±4	7691	2.815±0.003	2.817±0.003
0.5	7.2±0.5	2511±2	2467±2	188±4	7428	2.815±0.003	2.819±0.003
theoretical-affected water							
0.1	14.0±0.5	2509±2	2457±2	203±4	11401	2.815±0.003	2.812±0.003
0.2	8.5±0.5	2509±2	2463±2	194±4	11535	2.815±0.003	2.817±0.003
0.3	6.7±0.5	2511±2	2466±2	187±4	11600	2.815±0.003	2.819±0.003
0.4	5.7±0.5	2511±2	2469±2	185±4	11658	2.815±0.003	2.822±0.003
0.5	5.2±0.5	2511±2	2471±2	184±4	11691	2.815±0.003	2.822±0.003
changed-affected water							
0.1	–	2415±2	2349±2	110±4	156	2.744±0.003	2.729±0.003
0.2	–	2415±2	2356±2	110±4	284	2.744±0.003	2.732±0.003
0.3	–	2419±2	2363±2	119±4	279	2.744±0.003	2.739±0.003
0.4	–	2417±2	2354±2	110±4	412	2.744±0.003	2.729±0.003
0.5	–	2417±2	2361±2	117±4	425	2.744±0.003	2.732±0.003

[a] Approximate molality of DMSO (mol·kg^{-1}). [b] Affected number, equal to the number of moles of water affected by one mole of solute. [c] Band position at maximum (cm^{-1}). [d] Band position at gravity center (cm^{-1}). [e] Full width at half-height (cm^{-1}). [f] Integrated intensity (dm^{3}· mol^{-1} ·cm^{-2}). [g] The most likely O···O distance (Å). [h] Mean O···O distance (Å).

Table S2. The parameters of HDO band of NAGMA-affected water (m_{NAGMA} ~ 0.1 mol·kg^{-1}), DMSO-affected water, experimental and theoretical affected water and changed-affected water in NAGMA–DMSO systems, the bulk water, and the respective intermolecular oxygen–oxygen distances. R_{OO} errors have been estimated on the basis of the HDO bands position errors.

m_{DMSO} [a]	N [b]	ν^{o}_{OD} [c]	ν^{g}_{OD} [d]	*fwhh* [e]	I [f]	R^{o}_{OO} [g]	R^{g}_{OO} [h]
				bulk water			
	–	2509±2	2497±2	162±4	9953	2.827±0.003	2.844±0.003
				NAGMA			
	7.4±0.5	2505±2	2488±2	175±4	11107	2.827±0.003	2.834±0.003
				DMSO			
0.1	3.0±0.5	2514±2	2483±2	169±4	12161	2.821±0.003	2.831±0.003
0.2	3.0±0.5	2515±2	2483±2	169±4	12252	2.821±0.003	2.831±0.003
0.3	3.0±0.5	2515±2	2483±2	169±4	12222	2.821±0.003	2.831±0.003
0.4	3.0±0.5	2515±2	2483±2	169±4	12212	2.821±0.003	2.831±0.003
0.5	3.0±0.5	2515±2	2483±2	169±4	12202	2.821±0.003	2.831±0.003
				experimental-affected water			
0.1	9.5±0.5	2513±2	2487±2	172±4	11762	2.827±0.003	2.834±0.003
0.2	7.3±0.5	2513±2	2485±2	169±4	9952	2.827±0.003	2.834±0.003
0.3	7.2±0.5	2513±2	2485±2	169±4	8639	2.824±0.003	2.834±0.003
0.4	6.8±0.5	2513±2	2485±2	169±4	8207	2.824±0.003	2.834±0.003
0.5	6.6±0.5	2513±2	2485±2	169±4	7990	2.824±0.003	2.834±0.003
				theoretical-affected water			
0.1	10.2±0.5	2515±2	2486±2	175±4	11436	2.823±0.003	2.833±0.003
0.2	6.6±0.5	2515±2	2485±2	172±4	11647	2.823±0.003	2.833±0.003
0.3	5.4±0.5	2515±2	2485±2	172±4	11747	2.823±0.003	2.833±0.003
0.4	4.8±0.5	2515±2	2484±2	172±4	11818	2.823±0.003	2.833±0.003
0.5	4.4±0.5	2515±2	2484±2	172±4	11874	2.823±0.003	2.833±0.003
				changed-affected water			
0.1	–	2511±2	2496±2	139±4	563	2.836±0.003	2.839±0.003
0.2	–	2507±2	2493±2	147±4	410	2.836±0.003	2.836±0.003
0.3	–	2505±2	2493±2	153±4	311	2.827±0.003	2.834±0.003
0.4	–	2504±2	2494±2	158±4	304	2.822±0.003	2.834±0.003
0.5	–	2502±2	2495±2	155±4	292	2.819±0.003	2.836±0.003

[a] Approximate molality of DMSO (mol·kg^{-1}). [b] Affected number, equal to the number of moles of water affected by one mole of solute. [c] Band position at maximum (cm^{-1}). [d] Band position at gravity center (cm^{-1}). [e] Full width at half-height (cm^{-1}). [f] Integrated intensity (dm^{3} · mol^{-1} ·cm^{-2}). [g] The most likely O···O distance (Å). [h] Mean O···O distance (Å).

Table S3. Experimental and theoretical numbers of affected water molecules (N) for peptide–DMSO systems

$n_{DMSO.}/n_{pept.}$ [a]	$N_{exp.}$ [b]	$N_{DMSO,\ theor.}$ [c]	$N_{pept.,\ theor.}$ [c]	$N_{theor.}$ [d]	ΔN [e]
diglycine–DMSO system					
diglycine	11.3±0.5	–	11.3±0.5	11.3±0.5	
1.0	16.2±0.5	3.0±0.5	11.0±0.5	14.0±0.5	+2.2±0.5
2.0	10.5±0.5	3.0±0.5	5.5±0.5	8.5±0.5	+2.0±0.5
3.0	8.8±0.5	3.0±0.5	3.7±0.5	6.7±0.5	+2.1±0.5
4.0	7.7±0.5	3.0±0.5	2.7±0.5	5.7±0.5	+2.0±0.5
5.0	7.2±0.5	3.0±0.5	2.2±0.5	5.2±0.5	+2.0±0.5
DMSO	3.0±0.5	3.0±0.5		3.0±0.5	
NAGMA–DMSO system					
NAGMA	7.4±0.5	–	7.4±0.5	7.4±0.5	
1.0	9.5±0.5	3.0±0.5	7.2±0.5	10.2±0.5	–0.7±0.5
2.0	7.3±0.5	3.0±0.5	3.6±0.5	6.6±0.5	+0.7±0.5
3.0	7.2±0.5	3.0±0.5	2.4±0.5	5.4±0.5	+1.8±0.5
4.0	6.8±0.5	3.0±0.5	1.8±0.5	4.8±0.5	+2.2±0.5
5.0	6.6±0.5	3.0±0.5	1.4±0.5	4.4±0.5	+2.2±0.5
DMSO	3.0±0.5	3.0±0.5		3.0±0.5	

[a] The molar ratio of DMSO to peptide (approximate values). [b] Experimental values of N number in peptide–DMSO systems. The parameter was obtained in relation to the solute in excess (i.e., DMSO). [c] Theoretical N number of the pure component (DMSO or peptide) in the theoretical spectrum. It was calculated per one mole of DMSO (from a DMSO point of view). [d] The theoretical N number, if the molecules would not interact with each other in any way ($N_{DMSO,\ theor.} + N_{pept.,\ theor.}$). [e] The difference between experimental and theoretical N numbers in peptide–DMSO systems ($N_{exp.} - N_{theor.}$).

S3. Results of DFT calculations

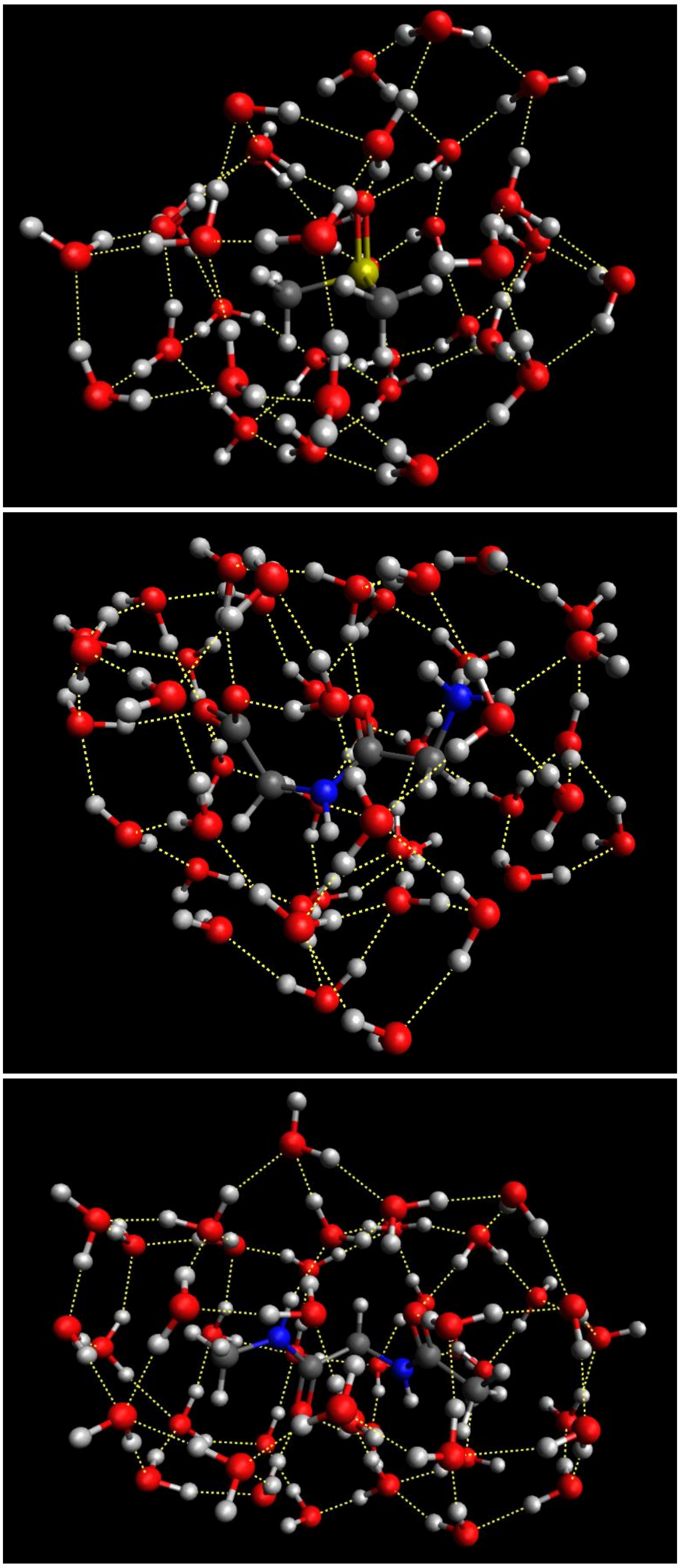

Figure S7. Structures of the DMSO—35H_2O, diglycine—42H_2O, and NAGMA—42H_2O systems, respectively, optimized using the DFT/ONIOM method.

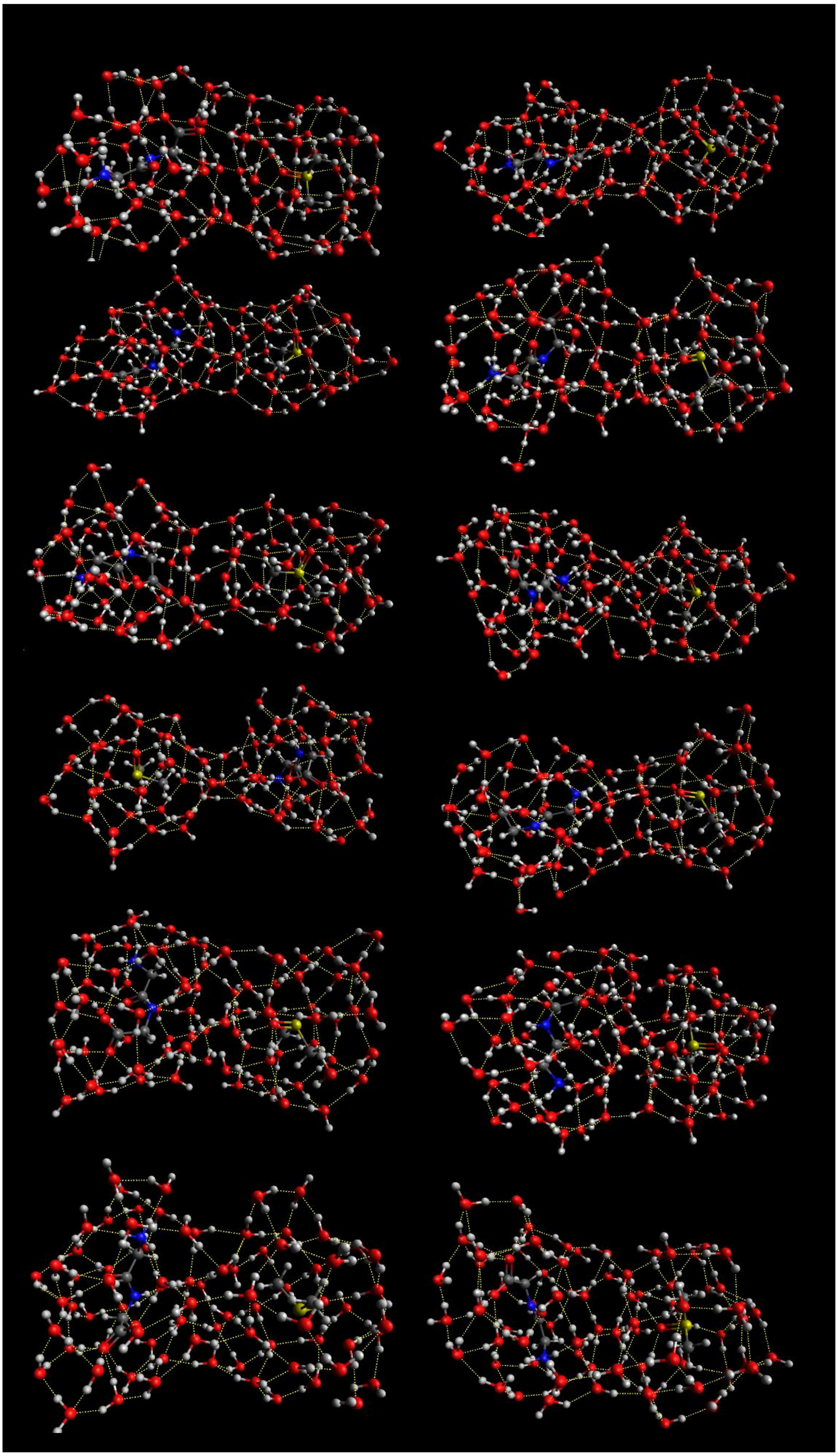

Figure S8. Structures of supercomplexes of diglycine—42H_2O:DMSO—35H_2O systems, respectively, optimized using the DFT/ONIOM method.

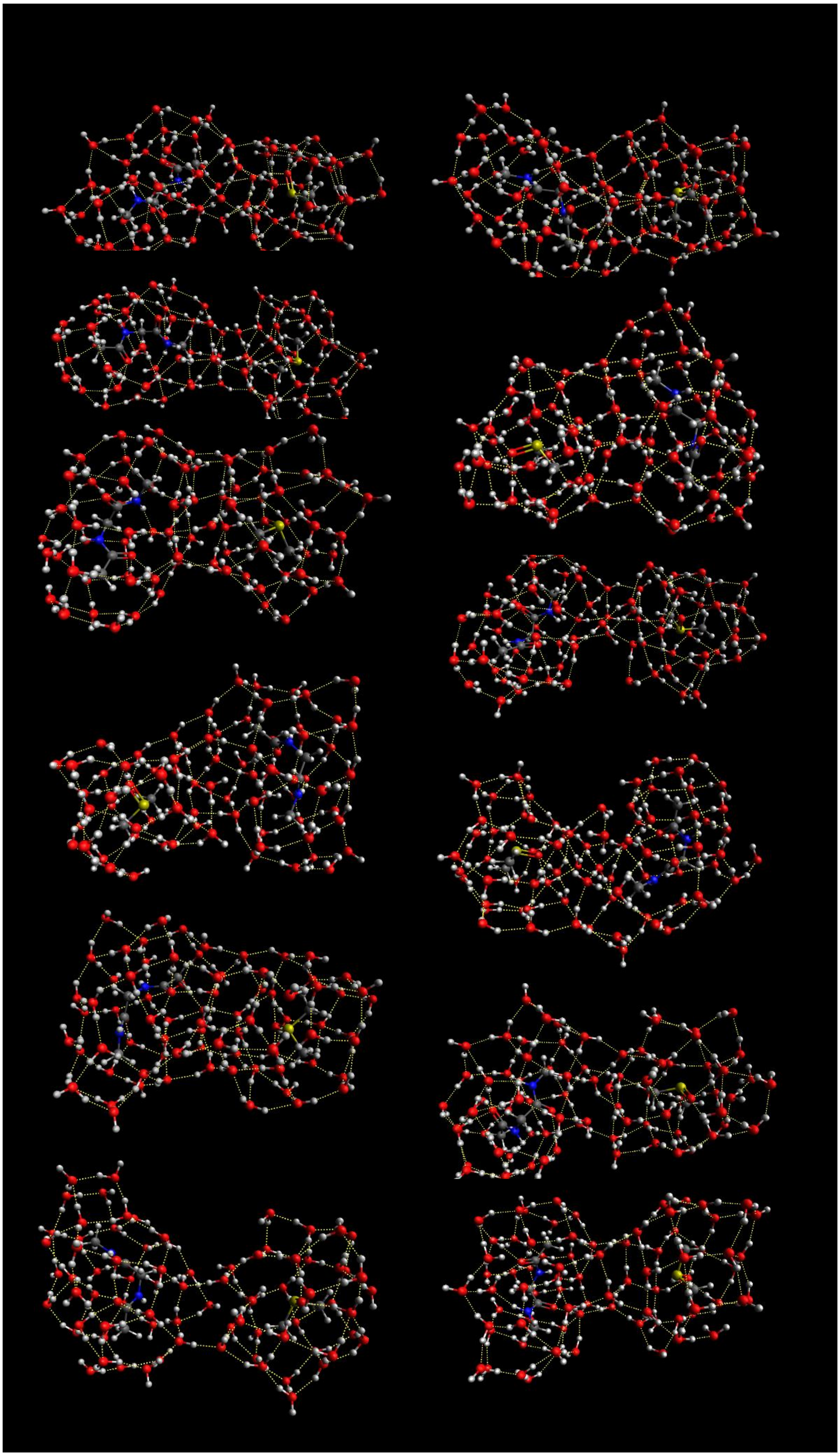

Figure S9. Structures of supercomplexes of NAGMA—42H_2O:DMSO—35H_2O systems, respectively, optimized using the DFT/ONIOM method.
